# Demonstration of facilitation between microalgae to face environmental stress

**DOI:** 10.1038/s41598-019-52450-9

**Published:** 2019-11-05

**Authors:** Emna Krichen, Alain Rapaport, Emilie Le Floc’h, Eric Fouilland

**Affiliations:** 1 MARBEC, Univ. Montpellier, CNRS, IFREMER, IRD, Sète, France; 2 MISTEA, Univ. Montpellier, INRA, SupAgro, Montpellier, France; 3 ADEME, Agence de l’environnement et de la Maîtrise de l’Energie, 20 avenue du Grésillé, BP 90406, 49004, Angers, Cedex 01 France

**Keywords:** Biotechnology, Ecology, Microbiology

## Abstract

Positive interactions such as facilitation play an important role during the biological colonization and species succession in harsh or changing environments. However, the direct evidence of such ecological interaction in microbial communities remains rare. Using common freshwater microalgae isolated from a High Rate Algal Pond *HRAP* treating wastewaters, we investigated with both experimental and modeling approaches the direct facilitation between two algal strains during the colonization phase. Our results demonstrate that the first colonization by microalgae under a severe chemical condition arose from the rapid growth of pioneer species such as *Chlorella sorokiniana*, which facilitated the subsequent colonization of low growth specialists such as *Scenedesmus pectinatus*. The pioneer species rapidly depleted the total available ammonia nitrogen favoring the specialist species initially inhibited by free ammonia toxicity. This latter species ultimately dominated the algal community through competitive exclusion under low nutrient conditions. We show that microbial successions are not only regulated by climatic conditions but also by interactions between species based on the ability to modify their growth conditions. We suggest that facilitation within the aquatic microbial communities is a widespread ecological interaction under a vast range of environmental stress.

## Introduction

One of the major challenges in microbial ecology is to understand the dynamics of communities of interacting species. Understanding the biological interactions and the time scales over which they occur is necessary to interpret the results of the directional succession process of communities’ development in the natural environment and artificial ecosystems. In aquatic systems, microalgae are present in natural waters such as oceans, lakes, rivers, and ponds and play a prominent role in the marine and fresh-water ecosystems where they drive major ecosystem processes. Strong similarities exist between marine and freshwater phytoplankton ecology^1^ when they face similar changes in growth conditions leading to temporal species succession. Abiotic forcing and biotic interactions can both result in successional trends in phytoplankton. The scientific discussion around the phytoplankton growth periodicity and succession has been dominated by the role of the environmental drivers including global climatic change (e.g. light, temperature, wind)^2–6^ local hydrological variations^7,8^ biological disturbances such as species invasion^9^, and chemical effects such as toxic pollutants, nutrient enrichment, or change in *pH* (see references^6,7,10–12^). On the other hand, the conditions governing phytoplankton growth over the seasonal change in plankton communities have mostly been discussed in the context of exploitative competition (e.g. Tilman, 1982)^13^ or algae-grazer interactions (e.g. Porter, 1977)^14^.

The competition for limiting nutrients is an important factor explaining phytoplankton species temporal successions. In marine ecosystems, small-cell diatoms usually grow rapidly in the first stage after a strong nutrient enrichment because of their higher growth rates and are then followed by larger-cell diatoms and dinoflagellates, which are more likely to occur when nutrients are depleted^15,16^. Similarly, the seasonal patterns of succession in freshwater ecosystems might be explained by the first occurrence of invasive small-sized species^17^, which can be expected to continue to expand until they either run out of nutrient or light energy or are controlled by zooplankton grazing^17,18^. These pioneer invasive species can be replaced by other phytoplankton species more prone to grow under nutrient depletion because of mixotrophy ability or mobility allowing them to exploit patches of nutrients not available to other microalgae^17^.

The ability to colonize a specific habitat usually explains the dominance and succession under changes in environmental conditions. For instance, changes in the algal assemblage in natural biofilm communities have been reported in the context of ecological succession that may be related to the population’s tolerance to the physical architecture of the developing mat or the resource limitations within the mat occurring as the biofilm develops^19^.

Positive interactions (i. e. facilitation) between organisms can occur when one organism makes the local environment more favorable for another either directly (such as by reducing thermal, water or nutrient stress via shading or through nutritional symbioses) or indirectly (such as by removing competitors or deterring predators)^20^. Positive feedbacks are the main driving biotic mechanism in plant community succession, particularly under harsh environmental conditions including physical or biotic stresses^21^ and are potentially important in aquatic systems influencing the dynamics of populations and communities^19,20^. However, fewer studies have discussed the role of positive interactions in aquatic microbial communities’ organizations. During the biofilm development, it was suggested that early stages of diatoms succession follow the “facilitation” model outlined by Connell and Slatyer^22^ when the extracellular mucilage production modified the physical biofilm characteristics and then enhanced the probability of successful immigration of some species more than others^23^. Similarly, it was suggested that algal mucilage and stalks within the biofilm facilitate periphyton development by encouraging cell surface adhesion and providing increased sites for colonization^24^.

Phytoplankton can substantially change its surrounding conditions of growth by increasing *pH* due to the uptake of inorganic carbon during photosynthesis^25^, decreasing transparency with the increase of biomass concentration^26^ or depleting key nutrients. Hence, we suggest that this phytoplankton-driven environmental modification can provoke shifts in assemblages of species, thus leading to successions. We suppose that under highly polluted conditions, similar to strongly anthropized ecosystems, an assemblage of typical pioneer species will first develop because of their potential for rapid dispersal and growth. We hypothesize that species showing the fastest growth rates and the strongest stress tolerance to harsh environments will be able to grow under such conditions, making the ecosystem more favorable for species that are more competitive in stable growth conditions through ecological facilitation.

Previous results from a study investigating the biological succession within *HRAP*s used for wastewater treatment showed the growth of the rapid-growing species *Chlorella sp*. followed by the slow-growing and grazing-resilient species *Scenedesmus sp*.^27^. Similar successions have been observed in other studies using *HRAP*s as well^28,29^. The successional trends of typical microalgal species growing in *HRAP*s have generally been interpreted as responses to predation and/or seasonal factors^28,30^. Based on the previous observations of dominant species dynamics^27^, we tested in this study the hypothesis that during the colonization phase of *HRAP*s supplemented with wastewaters, *Chlorella sp*. can modify its habitat and facilitate the growth conditions for *Scenedesmus sp*. by reducing the nutrient stress modulated by ammonia toxicity^31^. We suggest that microbial successions might not be regulated by climatic conditions only, but also through positive interactions between species facing external chemical stress. We conducted sets of laboratory experiments using the species molecularly identified as *C. sorokiniana* and *S. pectinatus* on isolates taken from the *HRAP* located in northern France during its colonization by *Chlorella sp*. and *Scenedesmus sp*. being both previously identified by microscopy^27^. The objectives of these experiments were to determine the inhibiting factor among ammonium ion $N{H}_{4}^{+}$, *pH* and free ammonia *NH*_3_ and to determine their respective effects on the growth rates of each species. Then, we used a modeling approach to test the magnitude of facilitation/competition on the two microalgae and, further, to explain the observed patterns in *HRAP* continuously supplemented with wastewater^27^. We also studied the resilience and succession times, providing informative proxies on the efficiency of the ecological facilitation and the successional trends depending on the initial populations’ densities. Our results supported the theoretical considerations of ecological facilitation between one tolerant and one sensitive organism to a gradient of resource toxicity/bioavailability.

## Results and Discussion

Three sets of experiments (denoted *SE*1, *SE*2 and *SE*3) were performed (*i*) to isolate the inhibitory effects of possible external factors such as high nitrogen concentrations or *pH* and (*ii*) to demonstrate a facilitation interaction between two species. We then show how to exploit the experimental data using a mathematical modeling approach, providing new insights on the facilitation phenomenon.

### No direct toxic effect of high $N{H}_{4}^{+}$ and *pH* on microalgae growth rates

**Figure 1:**
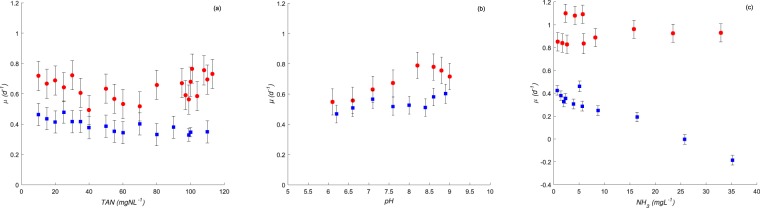
Growth rates from multiple comparisons on growth rate values estimated based on *ANOCOVA* analyses for *C. sorokiniana* (in red) and *S. pectinatus* (in blue) at different levels of (**a**) *TAN* concentrations, (**b**) *pH* conditions, (**c**) *NH*_3_ concentrations.

Chemical factors such as Total Ammonia Nitrogen *TAN* and *pH* can affect the rate and efficiency of photosynthesis of microalgae^32–34^. Negative effects of *TAN* (referring to nitrogen in two distinct forms: $N{H}_{4}^{+}$ and *NH*_3_ on algal growth and physiology might occur and vary significantly within classes of microalgae and within species (see^35^). The photosynthesis of different species of marine diatoms was severely inhibited at *TAN* concentrations in the range of 0.5 to 11 *mgN*.*L*^−1^(see^36,37^). At low *pH* values (<8), toxicity is likely associated with $N{H}_{4}^{+}$, while at alkaline *pH* values (>8), cell growth inhibition is rather due to *NH*_3_. During the first set of experiments *SE*1, the potential toxicity of high $N{H}_{4}^{+}$ was investigated for the two isolated algal species (molecularly identified as *C. Sorokiniana* and *S. pectinatus*) when *pH* values were adjusted to 7.5 at 25 °*C*. Hence, under such conditions, we ensure that 98% of *TAN* (ranging from 10 to 110 *mgN*.*L*^−1^) was present as $N{H}_{4}^{+}$ form (see the relative proportion of *NH*_3_ and $N{H}_{4}^{+}$ as a function of *pH* at 25 °*C*^38^). Under this range of concentrations, no significant difference in the growth rates of *S. pectinatus* or *C. sorokiniana* was measured (*p* > 0.05, *ANOVA* from *ANOCOVA* test results for four observations; see Fig. 1(a)). Therefore, the $N{H}_{4}^{+}$ form at such concentrations, typically found in wastewaters, did not affect the growth rates of both microalgae. Similarly, it was reported that species such as *Chlorella* are very tolerant to high *TAN* concentrations (max. 140 and 250 *mgN*.*L*^−1^ stated respectively in Collos and Harrison^35^ and Tam and Wong^39^). However, Przytocka-Jusiak *et al*.^40^ reported that cell division of *C. vulgaris* was inhibited at greater *TAN* concentrations (>300 *mgN*.*L*^−1^). Studies performed on *S. acuminatus* showed that cell growth was inhibited only when $N{H}_{4}^{+}$ concentrations were higher than 200 *mg*.*L*^−1^ (see reference^41^). Interestingly, it has been previously reported that algal photosynthesis of *S. obliquus* was inhibited at *TAN* above 28 *mgN*.*L*^−1^ if the culture *pH* exceeded 8.0 (see ref.^32^).

Because *pH* can vary during algal growth in ecosystems due to the rapid and large *CO*_2_ consumption of microalgae, this might directly or indirectly affect algal growth rates. The optimal *pH* of many freshwater algae is about 8 (see ref.^42^). The growth of many algal species is inhibited in waters at *pH* greater than 8 (reduction of productivity of *Chaetoceros sp*. and *Chlorella sp*. by 22 % when *pH* was raised from 8 to 9), while other species can grow well above *pH* 8 (e.g. *Amphora sp*. and *Ankistrodesmus sp*. at *pH* 9 and 10, respectively)^43^. High *pH* conditions limit the availability of *CO*_2_ while $HC{O}_{3}^{-}$ dominates, and then algae cannot efficiently accumulate carbon and require a high supply of carbonates for maintaining photosynthetic activity^42^ or reducing the affinity to free *CO*_2_ ^44,45^. During the second set of experiments *SE*2, the direct effect of *pH* was tested using *pH* values ranging from 6 to 9 on algal growth under low initial *TAN* concentration of about 1 *mgN*.*L*^−1^. As shown in Fig. 1(b), the tested *pH* conditions had no significant effect on the growth rates measured for both species, *S. pectinatus* and *C. sorokiniana* (*p* > 0.05, *ANOVA* from *ANOCOVA* test results for three replicates and three observations). Then, similar to high values of $N{H}_{4}^{+}$, the results did not support the hypothesis of a negative effect of high *pH* values on the growth rates of both studied species when cultured in medium containing 1 *mgN*.*L*^−1^ of *TAN* concentrations. Similarly, Azov and Goldman^44^ suggested that *pH* did not play a role in the magnitude of inhibition but the degree of dissociation of nontoxic $N{H}_{4}^{+}$ to toxic *NH*_3_. In other words, the dissociation of *TAN* as a function of *pH* is the main determinant of how much *NH*_3_ is available to inhibit photosynthesis. We suggest that *NH*_3_ concentrations in *SE*1 and *SE*2 were likely too low (<2 *mg*.*L*^−1^) to exhibit algal growth inhibition. Therefore, the effect of a broader range of *NH*_3_ concentrations was then tested on both species in the third set of experiments.

### Evidence of species-dependent ammonia effect

*NH*_3_ is considered the *TAN*’s most toxic form for aquatic organisms^46^. The third set of experiments *SE*3 was then performed on the same algal isolates (*C. Sorokiniana* and *S. pectinatus*) to test their growth under *NH*_3_ concentrations ranging from 0.56 to 29.42 *mgN*.*L*^−1^. The results for the growth rates of both isolates (represented in Fig. 1(c)) showed that the growth rates of *C. sorokiniana* measured under the different *NH*_3_ concentrations were similar (*p* > 0.05, *ANOVA* from *ANOCOVA* test results for three replicates and three observations). However, the growth rates of *S. pectinatus* were significantly different (*p* < 0.05, *ANOVA* from *ANOCOVA* test results for three replicates and three observations) with an important reduction in growth rates when *NH*_3_ exceeded 8.7 *mg*.*L*^−1^. Similarly, early works reported that *NH*_3_ at concentrations greater than 15 *mgN*.*L*^−1^ and at *pH* values over 8 inhibited the photosynthesis and growth of *S. obliquus*^31,44^. The resistance of *C. sorokiniana* to very high *NH*_3_ concentrations (362 *mg*.*L*^−1^) was previously reported^47^, suggesting that species can also adapt their metabolism and becoming more tolerant to high *NH*_3_ environments over time^31,40^.

In *HRAP*s initially supplemented with high *TAN* concentrations, *NH*_3_ toxicity is therefore expected to be associated with elevated *pH* due to intense photosynthetic activity^31^ and could cause the depletion of microalgae culture or promote replacement with other tolerant species to face the prevailing stress. This feature should be magnified considerably during the summer as the conversion between $N{H}_{4}^{+}$ and *NH*_3_ is also temperature dependent^48^.

### Evidence of facilitation interaction through a modeling approach

A modeling approach was used to identify the growth characteristics of both studied species to predict their dynamics when they are growing together. Based on nutrient dynamics monitored in the *HRAP*^27^, we assumed that *TAN* was the sole limiting substrate driving the algal growth. Moreover, we considered that *NH*_3_ would have a direct inhibitory effect on cell growth, whose fraction is given by the following expression:

$$f(pH,T)=\frac{1}{1+{10}^{pKa(T)-pH}}$$

with $pKa(T)=0.09018+\frac{2727.92}{T+273.15}$ (established within the temperature range of 0 °*C*–50 °*C* and a *pH* range of 6.0 to 10.0 (see^48^).

**Figure 2:**
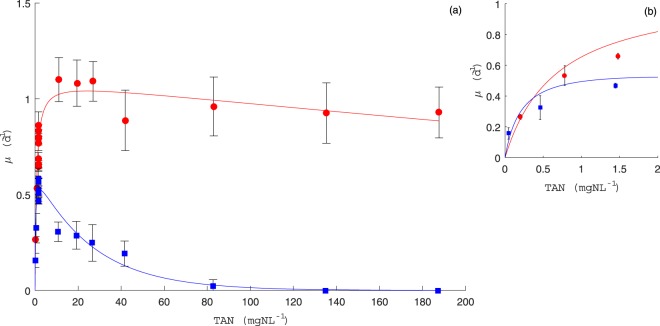
*SE*2 and *SE*3 data sets (full points) compared to the proposed kinetic model for *C. sorokiniana* (continuous red line) and *S. pectinatus* (continuous blue line) in (**a**), with a focus on low *TAN* concentrations in (**b**) (Data points are the mean of 3 duplicate measurements of growth rate).

As a first step, species growth rates were related to external *TAN* concentrations to calibrate one kinetic model, which could represent satisfactorily most of the data points of the previous test experiments obtained in both *SE*2 and *SE*3. The proposed model was inspired from Aiba-Edward’s model^49^ describing the substrate inhibition at high concentrations and consisting of a modified version of the Monod equation^50^, but here it has a slightly different mathematical expression as explained below. While Monod kinetics assumed that only one nutrient limits the growth of cells, the model we propose here includes that a by-product of this limiting nutrient (free ammonia nitrogen *NH*_3_−*N*) negatively affects cells growth as given by the following expression:

1 $$\mu (TAN,pH,T)=\hat{\mu }\frac{TAN}{k+TAN}{e}^{-\frac{TANf(pH,T)}{{k}_{i}}}$$

where $\hat{\mu }$ is the maximum growth rate (*d*^−1^), *k* is the affinity to substrate (*mg*.*L*^−1^), *k*_*i*_ is the inhibition constant of *NH*_3_−*N* (*mg*.*L*^−1^) and *f*(*pH*, *T*) is defined above. This growth function provided a good fit to experimental data describing the growth kinetics of the two species (see Fig. 2). The identified kinetic parameters are given in Table 1. From the fit of this kinetic model to data, the species *S. pectinatus* showed a strong affinity for nitrogen with a greater $\frac{\hat{\mu }}{k}$ ratio than that obtained for *C. sorokiniana* (see also the comparison of the two species kinetics with a particular focus on low *TAN* concentrations in Fig. 2(b)). In contrast, this latter species has a maximum growth rate (1.10 *d*^−1^) much higher than that of *S. pectinatus* (0.63 *d*^−1^). Consequently, *C. sorokiniana* would grow well at high *TAN* concentrations and would also tolerate high *NH*_3_ concentrations as reported by its highest inhibition constant (*k*_*i*_ = 79.82 *mgNH*_3_−*N*.*L*^−1^), while *S. pectinatus* would grow best at low *TAN* concentrations but would show a much faster decline in growth because of its high sensitivity to *NH*_3_ toxicity represented by a low *k*_*i*_ (2.25 *mgNH*_3_−*N*.*L*^−1^). Our results are in accordance with older chemostat experiments comparing *S. acutus* and *C. minutissima* under P-limited growth^51^.

**Table 1:** Calibration results on *SE*2 and *SE*3 growth data obtained in batch cultures.

Parameter	*C. sorokiniana*	*S. pectinatus*
$\hat{{\mu }}$ (*d*^−1^)	1.10	0.63
*k* (*mgN*.*L*^−1^)	0.68	0.22
*k*_*i*_ (*mgNH*_3_-*N*.*L*^−1^)	79.82	2.25
$\frac{\hat{\mu }}{k}$	1.62	2.85
*J* (least squares criterion)	0.08	0.04

The ecological succession of species presenting Monod- and Haldane-^52^ kind growth functions have already been shown theoretically^53^ but not yet experimentally. The Monod and Haldane kinetics were fitted to our data (results not shown). Their graphs closely resemble those given by (1), but with a higher least squares criterion *J*.

**Figure 3:**
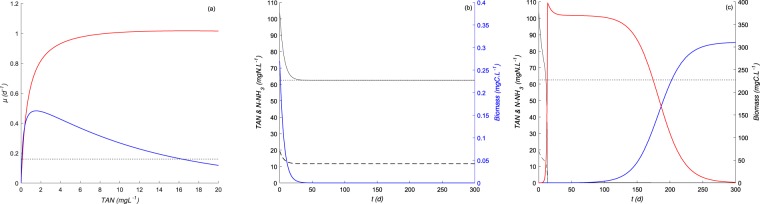
Simulation results obtained under a continuous supply with a high *TAN* concentration (dotted line in (**b,c**)) at a fixed dilution rate (dotted line in (**a**)). (**a**) growth functions previously identified for *C. Sorokiniana* (in solid red line) and *S. pectinatus* (in solid blue line), (**b**) dynamics when *S. pectinatus* is cultivated alone, (**c**) dynamics when *S. pectinatus* and *C. Sorokiniana* are cultivated together. In (**b,c**), the biomass variations over time are presented in blue for *Scenedesmus sp*. and in red for *Chlorella sp*. while the substrate variations are in solid black lines for *TAN* and in black dashed lines for *NH*_3_.

Knowing the growth performances of each species in the laboratory (Fig. 3(a)), we proposed a predictive model to explore how the assemblage of the two species might react under a fixed *pH* (8.6) and temperature (25 °*C*) in continuous culture to check if the hypothesis of ecological facilitation is verified. We used the initial conditions of substrate and biomass and the operational conditions (dilution rate and input substrate concentration) encountered in the previous study on *HRAP*s^27^. The results of our simulations are summarized in Fig. 3. These simulations revealed that *S. pectinatus* is unable to grow and is washed out because of the *NH*_3_ toxicity when cultivated alone under high *TAN* and *pH* conditions (see Fig. 3(b)). However, when both microalgae are introduced together under these latter conditions, *C. sorokiniana* grows rapidly first while the growth of *S. pectinatus* is inhibited because of high *NH*_3_. The rapid consumption of the nitrogen resource by *C. sorkiniana* induces low *NH*_3_ and less nitrogen availability, favoring the growth of the competitive *S. pectinatus* but not *C. sorokiniana* (see Fig. 3(c)). Therefore, the ecological facilitation between *C. sorokiniana* and *S. pectinatus* would be induced by *NH*_3_ toxicity and would explain their succession. These results also support the empirical evidence in plant communities that the balance between facilitation and competition can shift along an environmental gradient, with facilitation being successively more important in harsh environments^20^.

### Importance of the initial populations densities on the degree of facilitation in simulated *HRAP*

 Using the mathematical approach, we explored the influence of the initial densities of the studied species on some indicators of facilitation degree, which might be useful to further explore the optimization strategies for algal biomass production under high levels of ammonia stress in *HRAP*. There are different advantages to having a *Scenedesmus* dominance in an *HRAP* supplemented with wastewaters, as this species possesses a high affinity to nitrogen, is strongly resilient to predators^54^, and its biomass can be easily harvested^55^ and used for different purposes (e.g. lipid production^56^). For these reasons, we proposed to study theoretically some proxies of the facilitation efficiency such as resilience and succession times to provide information on the time required for the development of *S. pectinatus* in the *HRAP* under the previously stated operating conditions.

**Figure 4:**
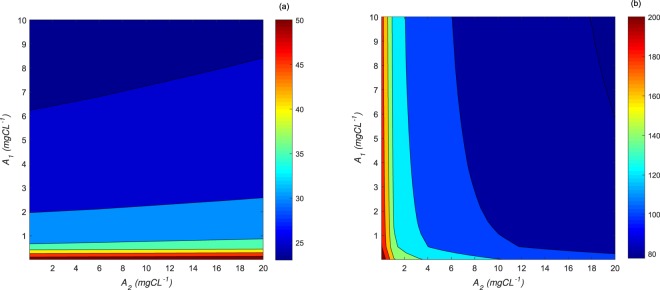
Isovalue diagrams: resilience time (**a**) and succession time (**b**) (in days) depending on the initial biomass densities of *C. sorokiniana* (*A*_1_) and *S. pectinatus* (*A*_2_) (in *mgC*.*L*^−1^) under a continuous supply of a high nitrogen concentration.

We defined the resilience time as the duration for *S. pectinatus* to reach its initial biomass value under the presence of the toxic *NH*_3_ concentrations. Moreover, because the succession of the two microalgae is required for maintaining *S. pectinatus* under high nutrient toxic levels, we also defined the succession time as the time for which the two species reach twice the same density level owing to the predominance of *S. pectinatus*. We plotted the iso-values of the resilience and succession times in the (*A*_1_, *A*_2_) plane, where *A*_1_, *A*_2_ are the initial densities of each species (see Fig. 4). These diagrams allow to see easily for which pairs of initial densities (*A*_1_, *A*_2_) one may expect low or high resilience or succession time (the shortest time is in blue, and the longest one is red). The diagram in Fig. 4(a) shows that the resilience time is more affected by the initial biomass concentrations of *C. sorokiniana* than that of *S. pectinatus*. This contrasts with the succession time (in Fig. 4(b)) which is more sensitive to the initial concentrations of *S. pectinatus*, especially when *C. sorokiniana* is initiated at concentrations values higher than 1 *mgC*.*L*^−1^. Such simulation would be of interest in further control of the diversity within an *HRAP* supplemented with wastewaters, especially for managing the periods of dysfunction (e. g. sudden algal crash, variations in wastewaters inflow). For example, it could be suggested to increase the initial concentration of *C. sorokiniana* (at a concentration higher than 6 *mg*.*L*^−1^) through bioaugmentation to ensure a rapid reduction of *NH*_3_ toxicity and rapid development of *S. pectinatus* in a minimum of 25 days. On the other hand, the time needed for *S. pectinatus* predominance over *C. sorokiniana* will depend on the initial concentration of *S. pectinatus* and the higher the *S. pectinatus*’s initial concentration would be (>12 *mg*.*L*^−1^) the faster the succession would occur (minimum of 80 days). Consequently, our theoretical results depicted with iso-value diagrams showed that the algal resilience and succession times within an intensive algal ecosystem are strongly dependent on the initial populations’ densities that may be used to control algal production processes in *HRAP*s.

### Validation of the ecological facilitation in real *HRAP*

**Figure 5:**
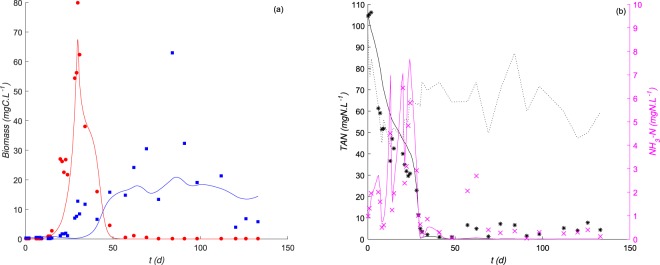
*HRAP* data points compared to the model prediction (in continuous lines) under a continuous supply of wastewater containing fluctuating concentrations of *TAN* (dotted black line). (**a**) Biomass variations over time of *Chlorella sp*. (in red) & *Scenedesmus sp*. (in blue), (**b**) substrate variations over time of *TAN* (in black) & *NH*_3_ (in magenta).

The dynamics of the biomass of *Chlorella sp*. and *Scenedesmus sp*. and the *TAN* concentrations measured in *HRAP* operating from 28 April 2015 to 8 September 2015 in Northern France^27^ were compared to model simulations (see Fig. 5) using the growth functions parameters of *C. sorokiniana* and *S. pectinatus* represented in Table 1. We made few changes to the initial model by adding mortality terms and considering different yields parameters values from those determined experimentally (all regarded as unknown constants) thus, still keeping the set of equations as simple as possible. The mortality terms were added to take into account the grazing effect on each algal species in an indirect way given the presence of predators in the *HRAP*. Changes in yield coefficients was requested knowing that heterotrophic bacteria were also growing in the pond and consuming nitrogen. The estimated parameters of yield and mortality coefficients obtained from the comparison of the model dynamics with data from *HRAP* are presented in Table 2. Assuming that higher predation pressure corresponds to a higher mortality coefficient, our results suggest that *Chlorella sp*. was likely more sensitive to grazing than *Scenedesmus sp*. known to produce a grazer-morphological defense according to a previous study^54^. However, the washout of *Chlorella sp*. at the system steady-state is probably not due to high pressure by a high mortality coefficient but rather to competition with *Scenedesmus sp*. when the environment becomes depleted of the nitrogen resource, as demonstrated through the previous simulation results in Fig. 3(c).

**Table 2:** Calibration results on *HRAP* data.

Parameter	*C. sorokiniana*	*S. pectinatus*
${y}^{\ast }$ ($gC/gN$)	2.81	0.30
${m}^{\ast }$ (*d*^−1^)	0.58	0.02
*J* (least squares criterion)	96.72	

We noted that the estimated yields coefficients in laboratory chemostat experiments after three days at steady-state (i. e. 5.9 ± 0.7 *gC*/*gN* and 5.0 ± 0.6 *gC*/*gN* for *C. sorokiniana* and *S. pectinatus*, respectively) were higher than those identified in the *HRAP* (see Table 2), which may be explained by the presence of denitrifying bacteria producing *N*_2_ subsequently lost through degassings^27^.

In the *HRAP*, the first bloom of *Chlorella sp*. happened at high concentrations of *NH*_3_ and has been replaced later by the bloom of *Scenedesmus sp*. (data in Fig. 5(a)). The distribution of these species was consistent with our experimental and modeling results on the species *C. sorokiniana* and *S. pectinatus* (see Fig. 3). This validates the importance of facilitation during the biological colonization of the *HRAP* under toxic levels of *NH*_3_. These results confirmed our initial hypothesis that the colonization of hypertrophic ecosystems by the stress-tolerant *Chlorella sp*. is a prerequisite for the development of the sensitive *Scenedesmus sp*. to *NH*_3_ toxicity. *Chlorella* is usually considered as an invasive phytoplankton or pioneer species because it maintains fast growth rates and assimilates resources with short generation times, and can dominate over slower-growing species^57,58^. In contrast, *Scenedesmus* is considered an affinity specialist^51^, and it can dominate in *HRAP*s over *Chlorella* and colonize the *HRAP*^27^ at low and nontoxic nutrient levels. Therefore, based on the model confrontation to real data in *HRAP*, pioneer organisms (here *Chlorella*) can modify their chemical environment by reducing ammonia toxicity, which can increase the fitness of the growth of sensitive and specialist organisms (here *Scenedesmus*).

Our study sheds a light on an ecological interaction within aquatic microbial communities that is rarely discussed in the literature, although it may explain ecological successions that occur without any visible external variations of growth conditions or mortality. Similarly to various types for macro-organisms^21^, the ability of aquatic microorganisms to drastically modify their immediate environment would impact the growth of neighbors. Because microalgae can change their light environment when growing (i. e. light attenuation by algal biomass), the growth of photoinhibited algal species can be facilitated by the biomass of other algal species less sensitive to photoinhibition^26^. In a similar way, under toxic metal stress, it has been suggested that the growth of *Cd*-sensitive microalgal species may be promoted by *Cd*-tolerant microalgal species reducing *Cd* in the media to low levels^59^. Therefore facilitation within the aquatic microbial communities through the reduction of inhibiting factors is likely to be a widespread interaction applied for a large range of environmental stress.

## Materials and Methods

### *HRAP* experiment

The pre-existing data used in this study were obtained in *HRAP* of 1.9 *m*^3^ working volume, continuously fed by pre-treated wastewaters (after screening and removing grit, sand, and grease) with a constant retention time of 6 days (see^27^). Algal blooms occurred naturally in the open pond without any algal inoculation. The period covered by this study was from 28 April 2015 to 8 September 2015. For the present study, we used the data obtained through analytical monitoring that was performed on the influent wastewater and on samples taken from the *HRAP*. They included water temperature, chemical analyses (*TAN* and *pH*), and the algal biomass of the two dominant algal species (*Chlorella sp*. and *Scenedesmus sp*., which were identified by microscopy) estimated using cell count from flow cytometry and converted into carbon units.

### Microalgae strains, cultivation conditions, and laboratory experiments

One Strain of freshwater microalgae *Chlorella sp*. and *Scenedesmus sp*. was isolated from the *HRAP* samples taken in October 2015. Individual strains were isolated in *Z*8 media^60^. The *Z*8 media was modified to *Z*8*NH*_4_ by replacing all nitrogen forms with ammonium salt (*NH*_4_*Cl*) as the sole source of nitrogen in the growth medium and by adding the *HEPES* buffer at 20 *mM*. The two species were maintained and cultivated under continuous light (100 *μEm*^−2^*s*^−1^) and temperature (25 °*C*). We performed three sets of experiments (*SE*1, *SE*2 and *SE*3) in batch reactors with a working volume of 40 *mL*. Each set was preceded by a pre-incubation phase in which the two species are preadapted to the fixed cultivation conditions in each set of experiments and providing sufficient fresh volumes for inoculations. The pre-incubations were performed either in continuous mode (in 2 *L* photobioreactors stirred at 300 *rpm*, one-sided illumined at 130 *μEm*^−2^*s*^−1^, before *SE*1) or in batch mode (in a shaken flask at 150 *rpm* of 200 *mL*, before *SE*2 and *SE*3). All batch experiments (in pre-incubation or in the three-test sets) were performed in laboratory incubators under a temperature set at 25 °*C* ± 2 °*C*, an orbital agitation at 150 *rpm* speed and an incident light intensity set at 50 *μEm*^−2^*s*^−1^ in *SE*2 and *SE*3.

The first pre-incubation in continuous photobioreactors were performed to determine the yield constants and provide sufficient fresh volumes for later inoculation in batch culture. Thus, each strain was growing for about 15 days until the biomass stabilization, under a constant temperature of 25 °*C* and a continuous supply of sterilized medium (*C*:*N*:*P* ratio at about 1:34:1) at a fixed *pH* value of 7.5 and a fixed dilution rate (0.25 *d*^−1^). After that, the growth of the two species was assessed in batch cultures (as described above) under different initial *TAN* concentrations ranging from 10 to 110 *mg*.*L*^−1^ keeping constant the concentrations of all other medium components. The *pH* value was maintained at 7.5 in all batch reactors of *SE*1.

In the second set of experiments *SE*2, prior to the experiment, the two species were pre-incubated in batch cultures for about 6 days in a sterilized medium of modified *Z*8*NH*_4_ with a *C*:*N*:*P* ratio at about 88:2:1 and a *pH* set at 7.5. Then, *SE*2 experiments were performed (as described above) under different *pH* conditions initially adjusted to 6.0, 6.5, 7.0, 7.5, 8.0, 8.4, 8.7, and 9.0 with *NaOH* or *HCl* while using a similar initial concentration of *NH*_4_*Cl* of 2 *mg*.*L*^−1^ that was supposed to be nontoxic for both microalgae strains.

Prior *SE*3, the 6-days pre-incubation of two species was performed in a sterilized medium of modified *Z*8*NH*_4_ with a *C*:*N*:*P* ratio at about 88:8:1 and a fixed *pH* value initially set at 8.6 (corresponding to the average value of *pH* measured in *HRAP*). Finally, in *SE*3, the growth of the two species was assessed in batch cultures under a large range of initial *NH*_4_*Cl* from 1.2 to 187.7 *mg*.*L*^−1^ keeping the *pH* at 8.6.

### DNA isolation, PCR, and sequencing

Genomic DNA was extracted from a 10 *mL* sample filtered onto a 0.2 *μm* membrane (PALL Supor 200 PES), using the standard phenol/chloroform method^61^. The 18S and ITS rDNA were amplified in PCR reactions using the Pfu polymerase (Promega) with the primers EAF3 (5′-TCGACAATCTGGTTGATCCTGCCAG-3′) and ITS055R (5′-CTCCTTGGTCCGTGTTTCAAGACGGG-3′)^62^. The PCR products were purified using the QIAquick Gel Extraction Kit (Qiagen) and sequenced using two primers (V4 Forward: 5′-AATTCCAGCTCCAATAGCGTATAT-3′ and ITS Forward: 5′-CCTTTGTACACACCGCCCGTCG-3′) to target specifically the variable V4 region of the 18S rDNA and the ITS region. Sanger sequencing was performed at Eurofins Genomics (GATC services).

### Sample analyses

The *pH* in each culture solution was determined daily (*pH* meter Symphony SP70P, VWR). For algal biomass estimation, samples were shaken to bring all the cells into suspension and subsamples were daily taken to measure absorbance. In *SE*1, the growth of algae was measured using optical density *OD* of the culture with a microplate reader (FLUOSTAR, BMG Labtech) at 650 *nm*. In *SE*2 and *SE*3, cell mass was measured by fluorescence (EX 450 *nm*, EM 680 *nm*) and *OD* at 650 *nm*, 730 *nm*, and 680 *nm* using a microplate reader (CHAMELEON, Hidex). Two different readers have been used due to a technical problem in CHAMELEON after the *SE*1 period.

In *SE*2 and *SE*3, subsamples were collected at the beginning and at the end of each experiment, for nutrient and biomass analysis. Samples were then filtered using (*i*) 0.2 *μm* Sartorius filters for measuring nutrients in filtrates and (*ii*) pre-combusted AE filters for measuring carbon biomass onto filters. Ammonia nitrogen was measured with a spectrophotometric test kit (SpectroQuant, Merck Millipore) and orthophosphate phosphorus according to an optimized molybdenum blue method^63^. After drying the filters (24 *h*, 60 °*C*), the particulate organic carbon representing mainly algal carbon biomass was analysed using an ANCA mass spectrometer (Europa Scientific).

### Data analysis

We performed the covariance analysis using the “aoctool” function of Matlab to compare significant differences in growth rates *μ* of algae after 48 *h* exposure at each tested condition. The technique required the grouped data of logarithm of the biomass *ln*(*x*) measured at time *t* (during the time period 0 to 48 *h*) for all tested condition. We modelled *ln*(*x*) as a linear function of *t* to determine whether the slope of the line, which represents an estimate of *μ*, varies among groups. Based on the model fit of the separate-lines model, the stats output structure from “aoctool” served as input to the multi-compare test “multcompare” function of Matlab, which allows for testing either slopes or intercepts.

### Modeling procedures

The first identifications of the growth function parameters for the two species were performed by fitting the proposed kinetic model (1) to the assessed values of specific growth rate data obtained in *SE*2 and *SE*3 for which cultivation conditions are either identical or different but would not be disruptive of the growth rates except for the initial *TAN* concentration. The optimal growth parameters were calibrated by the “fmincon” function of Matlab optimization toolbox used in minimizing a mean square criterion $J={\sum }_{i\mathrm{=1}}^{n}{({\mu }_{{i}_{exp}}-{\mu }_{{i}_{sim}})}^{2}$, where *μ*_*iexp*_ and *μ*_*isim*_ are the normalized experimentally estimated and model generated values of growth rates at the *i*^*th*^ experimental condition, and *n* is the total number of estimated growth rates corresponding to the total number of tested conditions *TAN* concentrations in *SE*2 and *SE*3.

Secondly, we used the identified growth functions on (1) to simulate the following system (2) in order to explore the species dynamics under a fixed *pH* (8.6) and temperature (25 °*C*) in a homogeneous continuous reactor.

2 $$\{\begin{array}{ccc}{\dot{A}}_{1} & = & ({\mu }_{1}(N)-D){A}_{1}\\ {\dot{A}}_{2} & = & ({\mu }_{2}(N)-D){A}_{2}\\ \dot{N} & = & -\,\frac{1}{{y}_{1}}{\mu }_{1}(N){A}_{1}-\frac{1}{{y}_{2}}{\mu }_{2}(N){A}_{2}+D({N}_{in}-N)\end{array}$$

This set of equations gives the variations over the time of both algal biomass of *C. sorokiniana* and *S. pectinatus* (in *mgC*.*L*^−1^) and substrate concentrations (*TAN* = *NH*_3_ + $N{H}_{4}^{+}$) (in *mgN*.*L*^−1^), denoted *A*_1_(*t*), *A*_2_(*t*) and *N*(*t*), respectively. The growth functions *μ*_1_(*N*) and *μ*_2_(*N*) depend only on *TAN* (when *T* = 25 °*C* and *pH* = 8.6, according to (1) and the parameters presented in Table 2), as the sole source of nitrogen supplied continuously at the fixed dilution rate *D* = 0.16 *d*^−1^ and the constant concentration *N*_*in*_ = 62.54 *mgN*.*L*^−1^ of wastewater encountered in the studied *HRAP*. The yield coefficients were taken equal to *y*_1_ = 5.93 *gC*/*gN* and *y*_2_ = 4.98 *gC*/*gN* for *C. sorokiniana* and *S. pectinatus*, respectively. These values were theoretically calculated from continuous photobioreactors experiments performed at pre-incubation for *SE*1 and given by ${y}_{i}=\frac{{A}_{i}^{\ast }}{{N}_{in}-{N}_{i}^{\ast }}$, where ${A}^{\ast }$ and ${N}^{\ast }$ are respectively the algal biomass and nitrogen concentrations at steady-state). The system (2) was solved using “ode23t” differential equation solver using the following initial conditions of substrate and biomass: *N*_0_ = 104.50 *mgN*.*L*^−1^, *A*_10_ = 0.0123 *mgC*.*L*^−1^ and *A*_20_ = 0.2698 *mgC*.*L*^−1^.

Under the same conditions as mentioned above, we theoretically studied the algal resilience and succession times as proxies of the facilitation efficiency for different initial biomass concentrations (in *mgC*.*L*^−1^) ranging between [0.0123, 10] and [0.05, 20] for *C. sorokiniana* and *S. pectinatus*, respectively. The iso-value diagrams were obtained using the “contourf” plot of Matlab.

Third, we validated the hypothesis of ecological facilitation on real dynamics in *HRAP*. We used the whole dynamics simulated over the time from the given initial condition until the system was at a quasi steady-stat and we compared data to the following model Eq. (3) including terms of mortality ${m}_{1}^{\ast }$ and ${m}_{2}^{\ast }$ on *A*_1_ and *A*_2_, respectively:

3 $$\{\begin{array}{ccc}{\dot{A}}_{1} & = & ({\mu }_{1}(N,pH,T)-D-{m}_{1}^{\ast }){A}_{1}\\ {\dot{A}}_{2} & = & ({\mu }_{2}(N,pH,T)-D-{m}_{2}^{\ast }){A}_{2}\\ \dot{N} & = & -\frac{1}{{y}_{1}^{\ast }}{\mu }_{1}(N,pH,T){A}_{1}-\frac{1}{{y}_{2}^{\ast }}{\mu }_{2}(N,pH,T){A}_{2}+D({N}_{in}-N)\end{array}$$

In this new set of Eq. (3), we considered the variations over the time of *N*_*in*_, *pH*, and *T* implemented into the model with interpolations performed between the real data points measured over time within the *HRAP*. We identified the unknown parameters (${m}_{1}^{\ast }$, ${m}_{2}^{\ast }$, ${y}_{1}^{\ast }$, and ${y}_{2}^{\ast }$) of the dynamic model using “fmincon” function of Matlab optimization toolbox. The optimal parameters assuring the best fit to data were constrained to be positive and defined in predefined intervals of boundary values after 100 consecutive estimations. Mortality constants were estimated within the interval [0, 1] (*d*^−1^), while yields coefficients were supposed to be ranged between *i*) minimal values theoretically calculated during the period of the dominance of each species in the *HRAP* (i. e. 1.3 ± 0.1 *gC*/*gN* and 0.5 ± 0.2 *gC*/*gN* for *Chlorella sp*. and *Scenedesmus sp*., respectively) and (*ii*) maximal values identified in our laboratory chemostat experiments after three days at steady-state (i. e. 5.93 ± 0.66 *gC*/*gN* and 4.98 ± 0.58 *gC*/*gN* for *C. sorokiniana* and *S. pectinatus*, respectively). The mean squared error was used as the criterion function for the model parameters estimation and was calculated as the square root of the variance of the observations (of *A*_1_, *A*_2_ and *N*) and divided by the number of measurements.
