# Author Correction: Demonstration of facilitation between microalgae to face environmental stress

**DOI:** 10.1038/s41598-020-59860-0

**Published:** 2020-02-21

**Authors:** Emna Krichen, Alain Rapaport, Emilie Le Floc’h, Eric Fouilland

**Affiliations:** 1 MARBEC, Univ. Montpellier, CNRS, IFREMER, IRD, Sète, France; 2 MISTEA, Univ. Montpellier, INRA, SupAgro, Montpellier, France; 3 ADEME, Agence de l’environnement et de la Maîtrise de l’Energie, 20 avenue du Grésillé, BP 90406, 49004 Angers, Cedex 01 France

Correction to: *Scientific Reports* 10.1038/s41598-019-52450-9, published online 05 November 2019

The original version of this Article omitted an affiliation for Emna Krichen. The correct affiliations for Emna Krichen are listed below:

MARBEC, Univ. Montpellier, CNRS, IFREMER, IRD, Sète, France

MISTEA, Univ. Montpellier, INRA, SupAgro, Montpellier, France

ADEME, Agence de l’environnement et de la Maîtrise de l’Energie, 20 avenue du Grésillé, BP 90406, 49004 Angers Cedex 01, France

Additionally, the Acknowledgements section contained typographical errors and was incomplete.

“We thank Elodie Lanouguère for microalgae isolation and cultivation, Ariane Atteia for identifying the microalgal strains, Martine Fortune for ammonia analyses, Patrick Raimbault for carbon content analyses, and Christine Felix for giving helping hand to experiments monitoring. We would also like to thank Bènèdicte Fontez for assistance with statistical analyses, and Jèrôme Harmand for fruitful comments and discussions during the MODEMIC research school on resource-consumer models (21–25 September 2015). This work was supported by the ADEME French Agency and the LabEx NUMEV incorporated into the I-Site MUSE funded by the French Research Agency (ANR) that have both funded the PhD grant of the first author. This study was also supported by the PHYCOVER project, which was funded by the French National Agency for Research (ANR-14-CE04-0011).”

now reads:

“We thank Elodie Lanouguère for microalgae isolation and cultivation, Ariane Atteia for identifying the microalgal strains, Martine Fortune for ammonia analyses, Patrick Raimbault for carbon content analyses, and Christine Felix for giving helping hand to experiments monitoring. We would also like to thank Bénédicte Fontez for assistance with statistical analyses, and Jérôme Harmand for fruitful comments and discussions during the MODEMIC research school on resource-consumer models (21–25 September 2015). This study benefited from the HRAP demonstrators developed and operated by SAUR. This work was supported by the ADEME French Agency and the LabEx NUMEV incorporated into the I-Site MUSE funded by the French Research Agency (ANR) that have both funded the PhD grant of the first author. This study was also supported by the PHYCOVER project, which was funded by the French National Agency for Research (ANR-14-CE04-0011).”

These errors have now been corrected in the PDF and HTML versions of the Article.

